# Post-Prescription Audit Plus Beta-D-Glucan Assessment Decrease Echinocandin Use in People with Suspected Invasive Candidiasis

**DOI:** 10.3390/medicina57070656

**Published:** 2021-06-26

**Authors:** Rita Murri, Sara Lardo, Alessio De Luca, Brunella Posteraro, Riccardo Torelli, Giulia De Angelis, Francesca Giovannenze, Francesco Taccari, Lucia Pavan, Lucia Parroni, Maurizio Sanguinetti, Massimo Fantoni

**Affiliations:** 1 Department of Laboratory and Infectious Diseases Sciences, A. Gemelli University Hospital Foundation IRCCS, 00168 Rome, Italy; brunella.posteraro@unicatt.it (B.P.); riccardo.torelli@policlinicogemelli.it (R.T.); giulia.deangelis@unicatt.it (G.D.A.); francesca.giovannenze@gmail.com (F.G.); francesco.taccari@policlinicogemelli.it (F.T.); maurizio.sanguinetti@unicatt.it (M.S.); massimo.fantoni@policlinicogemelli.it (M.F.); 2 Infectious Diseases Section, Department of Safety and Bioethics, Catholic University of the Sacred Heart, 00168 Rome, Italy; 3 A. Gemelli University Hospital Foundation IRCCS, 00168 Rome, Italy; saralardo@gmail.com; 4 Pharmacy Complex Operative Unit, A. Gemelli University Hospital Foundation IRCCS, 00168 Rome, Italy; alessio.deluca@policlinicogemelli.it (A.D.L.); lucia.pavan@policlinicogemelli.it (L.P.); lucia.parroni@policlinicogemelli.it (L.P.); 5 Department of Basic Biotechnology, Clinical Intensive Care and Perioperative Sciences, Catholic University of the Sacred Heart, 00168 Rome, Italy

**Keywords:** antifungal stewardship, *Candida* bloodstream infection, echinocandin

## Abstract

*Background and Objectives:* Overtreatment with antifungal drugs is often observed. Antifungal stewardship (AFS) focuses on optimizing the treatment for invasive fungal diseases. The objective of the present study was to evaluate the utility of a post-prescription audit plus beta-D-glucan (BDG) assessment on reducing echinocandin use in persons with suspected invasive candidiasis. *Materials and Methods:* This is a prospective, pre-post quasi-experimental study of people starting echinocandins for suspected invasive candidiasis. The intervention of the study included review of each echinocandin prescription and discontinuation of treatment if a very low probability of fungal disease or a negative BDG value were found. Pre-intervention data were compared with the intervention phase. The primary outcome of the study was the duration of echinocandin therapy. Secondary outcomes were length of hospital stay and mortality. *Results:* Ninety-two echinocandin prescriptions were reviewed, 49 (53.3%) in the pre-intervention phase and 43 (46.7%) in the intervention phase. Discontinuation of antifungal therapy was possible in 21 of the 43 patients in the intervention phase (48.8%). The duration of echinocandin therapy was 7.4 (SD 4.7) in the pre-intervention phase, 4.1 days (SD 2.9) in persons undergoing the intervention, and 8.6 (SD 7.3) in persons in whom the intervention was not feasible (*p* at ANOVA = 0.016). Length of stay and mortality did not differ between pre-intervention and intervention phases. *Conclusions:* An intervention based on pre-prescription restriction and post-prescription audit when combined with BDG measurement is effective in optimizing antifungal therapy by significantly reducing excessive treatment duration.

## 1. Introduction

Invasive candidiasis is becoming an emerging problem in hospital practice [1]. *Candida* is one of the leading causes of catheter-associated bloodstream infection (BSI) in the United States [2]. Mortality associated with invasive candidiasis is very high (51% in Internal Medicine in Italy) [3], and severely ill persons are more susceptible to *Candida* infections, leading to frequent antifungal drugs overtreatment. However, the diagnosis of invasive candidiasis and invasive fungal infection (IFI) is a complex task, because of the lack of standardized and widely accepted criteria. In addition, the large number of risk factors, limitations in diagnostic techniques, and lack of well-established criteria for initiating antifungal therapy are major problems in the approach to antifungal treatment. It follows that up to 42% of all antifungal therapies are empiric [4,5,6,7] and represent a huge field for potential optimization and antifungal stewardship (AFS) programs [8]. According to current definition, AFS refers to coordinated interventions to monitor and direct the appropriate use of antifungal agents in order to achieve the best clinical outcomes and minimize selective pressure and adverse events [9]. In some experiences, success in reducing overuse of antifungal therapies has been demonstrated, resulting in a containment of microbiological pressure on the ecosystem, which is recognized as the cause of resistance emergence [10]. In addition, the costs of antifungal therapies are often very high, and thus financial resource savings are an important driver of AFS. According to published guidelines [11,12], the optimal starting regimen for suspected invasive candidiasis are echinocandins, which mean high costs and potentially high selection pressure and induction of strains resistant to this crucial drug class. Thus, echinocandin discontinuation and de-escalation to azole-based therapies represents one of the cornerstones of any AFS programs.

Studies of beta-D-glucan (BDG) assessment have demonstrated its high negative predictive value [13,14]. Currently, BDG assessment has been widely introduced into clinical practice to discriminate people without candidemia and, when negative, offers valuable decision support for discontinuation of empiric antifungal therapy [13,15].

The objective of the present study was to evaluate the utility of a post-prescription audit plus BDG assessment on reducing echinocandin use in persons with suspected invasive candidiasis.

## 2. Materials and Methods

This is a prospective, pre-post quasi-experimental study of people starting echinocandins for suspected invasive candidiasis. The study was conducted at a 1100-bed university hospital in Italy (Fondazione Policlinico A. Gemelli IRCCS, Università Cattolica S. Cuore). An antimicrobial stewardship program has been active in the hospital since 2013; it is not active in hematological units and intensive care units (ICUs), and therefore patients in these units were not included in the study.

In our hospital, for an echinocandin treatment to be prescribed, a form is required from the hospital pharmacy. However, no restrictions were in place before the present intervention study, and every request was fulfilled. For fluconazole, the order is cumulative for the entire department, and the prescription is recorded on a nonelectronic form, and thus it was impossible to verify the prescription on a patient-by-patient basis. Therefore, patients who were prescribed fluconazole were excluded from the Intervention.

In the pre-intervention phase (Period 1), from January to May 2017, we reviewed all the echinocandins’ prescription forms sent to the hospital pharmacy service and we collected patients’ data. In the pre-intervention phase, we did not make any clinical suggestion, except when attending physicians actively requested an infectious diseases specialist advice. In the intervention phase (Period 2), from September 2017 to February 2018, we prospectively collected data of patients who were prescribed with echinocandins. Each echinocandin prescription was reviewed, IFI diagnosis was verified on both medical records and at the bedside, and inappropriate prescriptions were actively discussed with attending physicians who remained the final prescribers and could decide not to discontinue echinocandins. The intervention included discontinuation of treatment in patients with a very low probability of having IFI and with a negative BDG value, when available.

Risk factors for invasive candidiasis were considered according to a previous published article [16]: having a central venous catheter, total parenteral nutrition, recent surgery, previous admission to an ICU, previous antibiotic therapy, hemodialysis, solid organ transplantation, or multiple underlying medical conditions.

The primary outcome of the study was the duration of echinocandin therapy. Secondary outcomes were length of hospital stay (LOS) and mortality.

### 2.1. Microbiology

The Fungitell assay (Associates of Cape Cod, East Falmouth, MA, USA) was used to measure BDG. BDG results were evaluated using different cutoff values: 80, 200, 300, 400, and >500 pg/mL. For clinical use, the BDG test was considered positive if BDG had a result above the manufacturer’s recommended cut-off (80 pg/mL). Only BDG available within +48 h after initiation of antifungal therapy was considered for analysis. Informed consent was not required because the activity does not alter routine clinical practice, and only cumulative and anonymized data were analyzed.

### 2.2. Statistical Analysis 

Descriptive data are presented. Normally distributed values are expressed as mean (± standard deviation (SD)) and non-normally distributed values as median (interquartile range (IQR)). Chi-squared test or Fisher’s exact test were used to compare the distribution of categorical variables, and Student’s *t* or Mann–Whitney *U* test was used to compare quantitative variables. A two-sided *p* value < 0.05 was considered statistically significant. The Kaplan–Meier method was used to estimate the correlation between intervention and 30-day mortality. All statistical analyses were performed using SPSS 17.0 (IBM SPSS Statistics for Windows, SPSS Inc., Chicago, IL, USA).

## 3. Results

Since January 2017, all the echinocandin prescriptions (*n* = 92) were reviewed, 49 (53.3%) in the pre-intervention phase and 43 (46.7%) in the intervention phase. If a patient had more than one echinocandin prescription, only the first one was considered for the present study. The mean age of those enrolled was 67 years (SD 13.8), 31 (33.7%) were female, and 68 patients (73.9%) were admitted to a medical ward. None of the included patients were neutropenic (Table 1). Thirty-nine (42.4%) died within 30 days of starting antifungal therapy.

All the patients who began empirical antifungal therapy had clinical signs consistent with invasive candidiasis and at least one risk factor for invasive candidiasis.

None of the patients who received an empiric antifungal regimen were subsequently found to have culture-proved evidence of invasive candidiasis. No treatment was prescribed for prophylactic purposes. 

**Figure 1:**
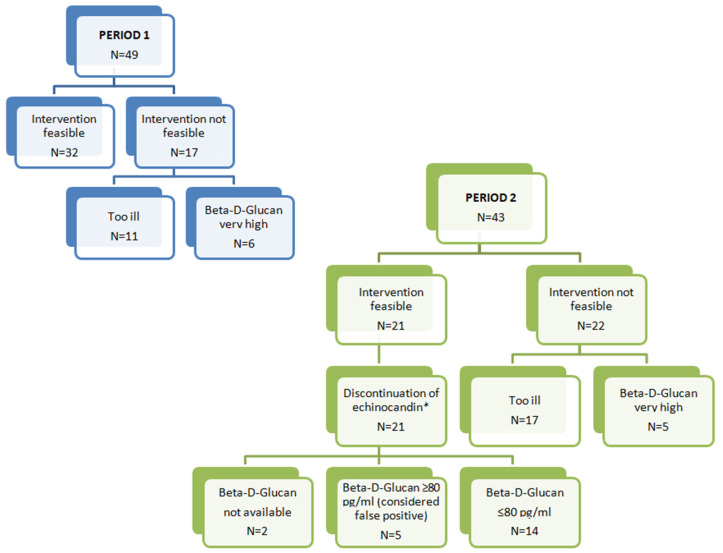
Flowchart of the study. * All patients who discontinued echinocandin had negative blood cultures for *Candida* spp.

Discontinuation of antifungal therapy was possible in 21 of the 43 patients in the intervention phase (48.8%). Of these 21 patients, BDG was available in 19 (in 14 cases, BDG was <80 pg/mL). In five cases, BDG >80 pg/mL was considered a false positive. In all patients who discontinued antifungal therapy, blood cultures (and peritoneal fluid cultures when indicated) were negative. Discontinuation of therapy was not possible in 22 cases: 17 patients were too sick, and in five cases, BDG was very high (>500 pg/mL) (Figure 1). The very high levels of BDG in these patients confirmed the suspicion of invasive candidiasis, contraindicating discontinuation of antifungal therapy, but it was not sufficient in the absence of positive cultures to establish a definite diagnosis of invasive candidiasis.

Individuals with BDG < 80 pg/mL or not done, when compared with patients with BDG > 80 pg/mL, had shorter duration of therapy (6.1 days (SD 5.0) versus 9.8 (SD 7.5); *p* = 0.007) and shorter length of stay (23.7 days (SD 31.4) versus 19.8 (SD 21.2); *p* = 0.06). The correlation between BDG outcome and duration of therapy was significant only in the intervention phase (Table 2).

Acceptance of the intervention from the treating physician, who remained the final prescriber and who could decide not to discontinue echinocandins, was very high, although this was not formally recorded. The requested time in terms of human resources was estimated at 1–1.5 days per week. 

The duration of echinocandin therapy was 4.1 days (SD 2.9) in persons undergoing the intervention, 8.6 (SD 7.3) in persons in whom the intervention was not feasible, and 7.4 (SD 4.7) in persons in the pre-intervention period (*p* at ANOVA = 0.016). The mean duration of therapy in persons who did not receive an intervention was 8.1 days (SD 6.3). Duration of therapy was shorter in the intervention group than in people who did not receive an intervention (*p* < 0.001). Length of stay was 19.1 days (SD 25.3) in persons who received the intervention, 21.2 days (SD 33.8) in persons in whom the intervention was not feasible, and 26.6 (SD 23.9) in persons in the pre-intervention phase (*p* at ANOVA 0.61) (Table 3).

Nine persons died in the intervention group (42.8%), 17 (34.7%) in persons in whom the intervention was not done, and 13 (59.1%) in persons for whom an intervention was not feasible (log-rank at Kaplan–Meier estimate = 0.64). 

## 4. Discussion

In the present study, we demonstrated that an intervention based on pre-prescription restriction and post-prescription audit when combined with BDG measurement is effective in optimizing antifungal therapy by significantly reducing excessive treatment duration. 

Several predictive scores of invasive fungal infections are available [17,18,19] and include variables that are largely present in most hospitalized patients or are very impractical to obtain in non-intensive care units, such as the number of *Candida* colonization sites. The predictive power of these variables is therefore poor. This uncertainty about the likelihood of diagnosis as well as awareness of the high mortality of invasive fungal infections likely underlies widely recognized overtreatment. In one study, more than half of antifungal prescriptions were described as suboptimal, with 16% considered unnecessary [6]. Overtreatment may be associated with several disadvantages. First, unnecessary pressure on the local ecology may contribute to increased antifungal resistance [10]. An increased incidence of *Candida* infections caused by strains resistant to fluconazole or to echinocandin has been reported [20,21,22]. In addition, many antifungals have complex pharmacokinetics and require careful consideration of drug–drug interactions [23]. Moreover, adverse events should be considered in the cost-effectiveness of any antifungal treatment [24]. Finally, antifungal treatments are often associated with high costs.

AFS programs are designed to reduce the overuse of empiric antifungal therapies and to optimize individualized regimens. The cornerstones of these programs are post-prescription audits, formulary restrictions (preauthorization strategies), discontinuation of therapies when they are not needed, and de-escalation from echinocandin to oral fluconazole when appropriate [8]. To date, published studies on the effectiveness of AFS programs are not numerous, and the most effective type of intervention remains unclear. Post-prescription audits, often associated with pre-prescription restrictions, have demonstrated a significant reduction in antifungal consumption [8,25,26,27,28]. In a Spanish study of 100 patients admitted to different hospital wards, a non-mandatory bedside intervention significantly reduced consumption and cost of antifungal treatments [29]. Whitney et al. published the results on a 6-year AFS program on more than 400 patients with a comprehensive approach that included stewardship rounds. The authors found a significant decrease in overall antifungal consumption and financial costs [29]. A few other studies have shown significant decrease in costs [26,30] and adverse events [31], without a different impact on survival. However, some of them identified a very small sample size [30,32], and most of them focused on optimizing only high-cost antifungal treatment [26]. Few studies incorporated therapeutic drug monitoring (TDM) measurement into AFS programs. 

Several papers have previously demonstrated a very high negative predictive value of BDG, and clinicians can use a negative result to discontinue empiric antifungal therapy. However, only one published study has shown results on the implementation of BDG within AFS programs [32]. In the present study, negative BDG results were used to discontinue antifungals in 14 of 43 patients in the intervention phase (32.5%). Considering only patients for whom an intervention was feasible, BDG was used to discontinue unnecessary antifungal treatments in 14 of 21 cases (66.7%). Because spending on antifungals in our hospital was approximately EUR 1,200,000, a 32.5% savings would allow us to reduce costs by 390,000 per year. The cost of the BDG test was less than savings. Moreover, it should be taken into account that the time needed to get BDG results is shorter (few hours, depending on laboratory’s capacity) than classic culture methods, thus representing a fundamental tool for early unnecessary antifungal discontinuation in AFS programs. 

In the present study, the duration of empiric therapy was longer for patients without an intervention. Possibly, without the intervention of the AFS team, noninfectious disease physicians are more reluctant to de-escalate or discontinue antimicrobial treatments. 

The overall 30-day mortality in the present study was 42.4%, similar to previously published studies [1,8], demonstrating that antifungal discontinuation was safe and did not result in unintended increased mortality.

Previously published studies included mainly patients with hematological diseases, and the most represented disease was aspergillosis. Hematological patients have peculiar characteristics such as the use of a high rate of antifungal prophylaxis, specific guidelines for antifungal treatment management, and noninfectious disease specialists as primary prescribers (so-called champions). In our study, hematologic patients were excluded, and results included, through a bedside approach, a hospital-level approach in a nonspecific setting.

It may be argued that fluconazole-treated patients should be included in AFS programs and that excluding them from this intervention may have reduced study size and statistical significance. However, after IDSA guidelines published in 2016 [12], echinocandins are considered the agent of choice in the suspicion of invasive candidiasis, even in non-neutropenic patients, and the use of fluconazole is restricted as an alternative agent in patients not critically ill and in those who have a low risk of fluconazole-resistant organisms. It follows that the use of fluconazole as empiric starting therapy in patients with suspected IFI has markedly decreased in clinical practice. For example, the use of echinocandins as initial antifungal therapy in *Candida* bloodstream infections at our institution has increased from 60.7% in 2013 to 88% in 2019 (unpublished data). Thus, despite its potential role in widening the pool of suitable patients for AFS, adding fluconazole-treated patients to this study population would not have had a great statistical impact. 

Nevertheless, it is reasonable that our intervention will be effective, even in patients receiving fluconazole as empiric antifungal therapy. Actually, in previous studies on AFS programs including fluconazole-treated patients, the majority of those who received empiric antifungal therapy had no diagnostic criteria for invasive fungal infection, and stopping antifungal therapy was one of the most commonly applied interventions [29].

Lack of staff time is one of the most frequent factors considered as a barrier to AFS, according to a UK survey [26]. However, time spent on the intervention in our experience has been limited and low-cost infrastructure is needed for the program. This suggests easy reproducibility of the intervention in other clinical centers. 

Our study has several limitations. First, the single-center design limits the generalizability of the results to hospitals with different patient populations. Second, because hematologic patients are not included, neutropenic patients are very rare, and no conclusions can be drawn for this population. Third, adverse effects of antifungals and readmission rates were not evaluated. 

## 5. Conclusions

In conclusions, we demonstrated significant resource savings through reduction in the duration of antifungal therapy by means of an easily reproducible intervention. AFS programs are feasible and cost-effective, especially when combined with the use of well-validated biomarkers such as BDG. AFS could be a standard of care in hospitals with specialized units as well as a reference point for noninfectious disease specialists.

## Figures and Tables

**Table 1:** Characteristics of the 92 patients for whom an echinocandin was prescribed.

Age, Mean (SD), Years.	67.0 (13.8)
Female (%)	31 (33.7)
Medical ward (%)	68 (73.9)
Surgical ward (%)	24 (26.1)
Anidulafungin	41 (44.6)
Caspofungin	51 (55.4)
Beta-D-glucan value	
<80 pg/mL	52 (56.5)
>80 pg/mL	27 (29.3)
>500 pg/mL	10 (10.9)
Not done	13 (14.1)

**Table 2:** Correlation between beta-D-glucan results with duration of therapy and length of stay.

**Duration of Antifungal Therapy, Days, Mean (SD)**	**Beta-D-Glucan < 80 pg/mL or not Done**	**Beta-D-Glucan > 80 pg/mL**	***p***
Total	6.1 (5.0)	9.8 (7.5)	0.007
Pre-intervention period	6.4 (4.9)	8.4 (5.9)	0.29
Intervention period	5.5 (5.2)	10.8 (7.9)	0.026
**Length of stay, days, mean (SD)**	**Beta-D-glucan <80 pg/mL or not done**	**Beta-D-glucan >80 pg/mL**	***p***
Total	23.7 (31.4)	19.8 (21.2)	0.06

**Table 3:** Outcomes of the study.

**Outcomes**	**Intervention not Done** ***N* = 71**		**Intervention Done *N* = 21**	***p***
Antifungal therapy duration, days, mean (SD)	8.1 (6.3)		4.1 (2.9)	<0.001
Length of hospitalization, days, mean (SD)	23.6 (29.8)		19.1 (25.3)	0.54
Death (%)	30 (42.3)		9 (42.8)	0.96
**Outcomes**	**Period 1** ***N* = 49**	**Period 2,** **not feasible** ***N* = 22**	**Period 2,** **feasible** ***N* = 21**	***p* at ANOVA**
Antifungal therapy duration, days, mean (SD)	7.4 (4.7)	8.6 (7.3)	4.1 (2.9)	0.016
Length of hospitalization, days, mean (SD)	26.6 (23.9)	21.2 (33.8)	19.1 (25.3)	0.61
Death (%)	17 (34.7)	13 (59.1)	9 (42.8)	0.57

## Data Availability

Data are available from the corresponding author upon request.
